# Overexpression of mitochondrial STAT3 protein improves colonic inflammation and fibrosis in inflammatory bowel disease by enhancing mitochondrial function

**DOI:** 10.3389/fimmu.2026.1728341

**Published:** 2026-04-13

**Authors:** A Ram Lee, Haeyoun Choi, Seon-Yeong Lee, Hye yeon Kang, Young-Mee Moon, Suk Woo Nam, Bo-In Lee, Mi-La Cho

**Affiliations:** 1 Lab of Translational ImmunoMedicine, Catholic Research Institute of Medical Science, College of Medicine, College of Medicine, The Catholic University of Korea, Seoul, Republic of Korea; 2 Department of Pathology, College of Medicine, The Catholic University of Korea, Seoul, Republic of Korea; 3 Lab of Mitochondrial Immunomedicine center for Global Health Therapeutics (MIGHT), Catholic Research Institute of Medical Science, College of Medicine, The Catholic University of Korea, Banpo-daero, Seocho-gu, Seoul, Republic of Korea; 4 Department of Biomedicine & Health Sciences, College of Medicine, The Catholic University of Korea, Seoul, Republic of Korea; 5 Department of Microbiology, College of Medicine, The Catholic University of Korea, Seoul, Republic of Korea; 6 Catholic Hematopoietic Stem Cell Bank, College of Medicine, The Catholic University of Korea, Seoul, Republic of Korea; 7 Functional RNomics Research Center, The Catholic University of Korea, Seocho-gu, Seoul, Republic of Korea; 8 Division of Gastroenterology, Department of Internal Medicine, College of Medicine, Seoul St. Mary’s Hospital, The Catholic University of Korea, Seoul, Republic of Korea

**Keywords:** gene therapy, inflammatory bowel disease, microbiome, mitochondria, mitochondrial STAT3

## Abstract

**Introduction:**

The STAT3 protein is involved in mitochondrial functions such as the respiratory electron transport chain, regulation of cellular metabolism, and scavenging of reactive oxygen species. Inflammatory bowel disease (IBD) is associated with damaged intestinal cells and mitochondrial dysfunction due to the inflammatory environment of the intestine. Here, we studied the potential use of the *Stat3* gene to induce STAT3 expression in mitochondria to help treat IBD.

**Methods:**

We transferred the *Stat3* gene and examined its effects on the expression of proinflammatory cytokines and fibrosis markers, and mitochondrial function, in intestinal tissues via immunohistochemistry. The microbiomes of mice were also analyzed.

**Results:**

The gene increased the expression of mitochondrial STAT3 (mtSTAT3), which reduced the levels of iNOS and fibrosis factors (aSMA, COL1A1) as well as proinflammatory cytokines (IL-17, IL-6) in the colon. It also enhanced mitochondrial function in the colon, and in immune cells, and led to higher levels of the beneficial bacteria *Lactobacillus reuteri* and *Akkermansia muciniphila* in the intestine. Taken together, these changes helped alleviate colitis and protected against intestinal damage.

**Discussion:**

Stat3 gene transfer targeting mtSTAT3 expression ameliorates colitis, enhances mitochondrial function in the colon, and reduces inflammation via inhibition of the inflammatory response and necroptosis, offering a potential treatment for IBD.

## Introduction

Inflammatory bowel disease (IBD) is a chronic and relapsing immune-mediated disease that leads to long-term impairment of the gastrointestinal tract. There are two main forms, ulcerative colitis (UC) and Crohn’s disease (CD) (1). IBD is a global health problem due to its increasing incidence and lifelong morbidity (2). Although poorly understood, risk factors include immune-mediated factors, microbiota, environmental factors, and genetic predisposition (3). In IBD, distorted barrier function of the intestinal epithelium allows bacteria to enter the mucosa and induce inflammation (4). The release of proinflammatory cytokines such as tumor necrosis factor TNF-α further damages the intestinal epithelium and increases permeability; this vicious cycle leads to chronic inflammation with elevated levels of proinflammatory cytokines (5, 6).

IBD is also associated with mitochondrial damage (7). Mitochondria are the predominant source of reactive oxygen species (ROS) in the cell as a byproduct of OXPHOS. While low levels of ROS are neutralized by antioxidant machinery under normal cellular conditions, damaged mitochondria produce excessive oxidative stress and can compromise epithelial barrier function and induce IBD (8). Structurally abnormal mitochondria are observed in the intestines of IBD patients, and in colitis mouse models, which show signs of mitochondrial malfunction including oxidative stress and decreased ATP production (9–13).

STAT3 is a transcription factor that mainly translocates into the nucleus and is associated with various cellular physiological functions (14). However, some of it localizes to mitochondria, referred to as mitochondrial STAT3 (mtSTAT3) (15). A growing body of evidence suggests that mtSTAT3 not only positively regulates the activity of the electron transport chain but also suppresses the production of mitochondrial ROS (mtROS) and enhances mitochondrial function (15, 16). For example, ROS levels are higher in the bone marrow of STAT3-deficient mice compared to wild-type mice. In addition, impaired mitochondrial function can be restored via mitochondria-targeted STAT3, resulting in a significant reduction in ROS production (15, 17). Nonetheless, the effect of mtSTAT3 on IBD has not yet been determined.

Therefore, in this study, we comprehensively evaluated the effects of inducing mtSTAT3 expression via transfer of the *Stat3* gene into various models under diverse conditions.

## Materials and methods

### Patients

Colon tissues from 12 patients diagnosed with IBD (UC subtype) were examined. Of the 12 patients, 6 were severe cases while 6 patients were in remission. Disease severity was determined based on the Mayo Endoscopic Score and fecal levels of calprotectin. Colonic mucosal tissue was obtained from the rectum during surveillance colonoscopy. This study was approved by the Institutional Review Board of Seoul St. Mary’s Hospital (XC18TEDI0027) and performed in accordance with the Helsinki II Declaration.

### Western blot

Anti-STAT3 (#9139; Cell Signaling Technology), anti-p727-STAT3 (#9134; Cell Signaling Technology), anti-Flag (#3033S; Cell Signaling Technology), anti-Tubulin (ab6161; Abcam), and anti-COX4 (#4844; Cell Signaling Technology) antibodies were used to detect the corresponding protein levels by Western blotting (SNAP i.d. Protein Detection System; Merck Millipore, SNAP2BHMB050). Protein concentrations were determined using the bicinchoninic acid assay (BCA; #23235; Thermo Fisher Scientific). Primary antibodies were diluted in 0.1% skim milk in Tris-buffered saline (DUCHEFA, T1501.5000) containing Tween-20 (SIGMA, P1379), and membranes were incubated with the primary antibodies for 15 min at room temperature. After washing, membranes were incubated with horseradish peroxidase-conjugated secondary antibodies for 10 min at room temperature. Band intensities were quantified by image-capture densitometry. Full-length, uncropped images of all Western blots are provided in **Supplementary Figure S1**.

### DSS-induced mouse model of colitis

Colitis was induced in male C57BL/6 mice (Orient Bio Inc., Gyeonggi-do, Korea) via oral administration of 3% DSS (MP Biomedicals, Santa Ana, CA, USA) mixed in drinking water for 5 days. The mice were randomly separated into two groups (n = 5 per group). The mice were intravenously injected with 100 μg mtSTAT3 overexpression vector (TG mice) or control vector in 200 μL saline, 1 day before colitis induction. Additional injections were given 4 and 9 days after induction. TG mice received 3% DSS for 5 days. Body weights were measured daily. All experimental procedures were approved by the Department of Laboratory Animals, Institutional Animal Care and Use Committee (IACUC) of the School of Medicine, Catholic University of Korea, and conformed with the guidelines of the United States National Institutes of Health (Permit Number: 2023-0104-03). All animals were euthanized using carbon dioxide (CO_2_) inhalation in accordance with the AVMA Guidelines for the Euthanasia of Animals. CO_2_ gas was introduced into the chamber at a controlled rate of 40% of the chamber volume per minute, following the displacement method recommended by the guidelines. Pre-filling of the chamber was avoided. Animals were monitored for complete cessation of respiration and heartbeat to confirm death.

### Histopathological analysis

The mice were euthanized and their colons were excised, fixed in 10% formalin, and embedded in paraffin. The embedded tissues were cut into sections (5 µm thick) using a microtome (Leica Biosystems, Wetzlar, Germany) and stained with hematoxylin and eosin (H&E). Histological damage was quantitatively assessed using a validated scoring system for DSS-induced colitis. Four parameters were evaluated: (i) Loss of epithelium (0: none; 1: 0–5%; 2: 5–10%; 3: >10%), (ii) Crypt damage (0: none; 1: 0–10%; 2: 10–20%; 3: >20%), (iii) Depletion of goblet cells (0: none; 1: mild; 2: moderate; 3: severe), and (iv) Infiltration of inflammatory cells (0: none; 1: mild; 2: moderate; 3: severe). The individual scores were summed to generate a total histological score ranging from 0 to 12. All evaluations were performed by two observers blinded to the experimental groups.

### Confocal microscopy

The tissue sections were stained for COX4 (NB110–39115; Novus, St. Louis, MO, USA), p727-STAT3 (ab32143; Abcam, Cambridge, UK), and DAPI (D3571; Invitrogen, Waltham, MA, USA); washed with phosphate-buffered saline (PBS); fixed in 4% paraformaldehyde; washed again with PBS; and blocked for 30 min with 10% normal goat serum. The expression of mtSTAT3 was estimated according to fluorescence intensity using an LSM 510-META confocal laser scanning microscope (Zeiss, Oberkochen, Germany).

### Immunohistochemistry

The sections were then incubated overnight at 4 °C with the following primary antibodies at a 1:200 dilution: anti-inducible nitric oxide synthase (iNOS) (ab15323; Abcam), anti-IL-17 (ab79056; Abcam), anti-IL-6 (NB600–1131; Novus), anti-ZO-1 (40-2200, Invitrogen), anti-COL1A1 (PA5-50938; Thermo Fisher Scientific, Waltham, MA, USA), and anti-aSMA (ab7817; Abcam). Then, the sections were incubated with secondary biotinylated antibodies for 30 min with streptavidin–peroxidase complex. The reaction products were developed using the chromogen 3,3-diaminobenzidine (K3468; Dako Corp., Carpinteria, CA, USA).

### Flow cytometry

Mesenteric lymph nodes (MLNs) were surgically harvested and mechanically dissociated by passing them through a 70um cell strainer in cold PBS. The resulting single-cell suspensions were washed and adjusted to a concentration of 1x 10^6 cells/mL in RPMI-1640 medium supplemented with 10% FBS. To analyze Th2 and Th17 populations, cells were stimulated with Cell Stimulation Cocktail (containing PMA, Ionomycin, and Brefeldin A) for 5 hours at 37 °C. Following stimulation, cells were first stained with anti-CD4 for surface markers. Subsequently, the cells were fixed and permeabilized using a Cytofix/Cytoperm kit (BD Biosciences) according to the manufacturer’s instructions before staining for intracellular IL-4 and IL-17.

Th2 and Th17 cells were stained for PC5.5-CD4 (#45-0042-82; eBioscience, Santa Clara, CA, USA), PE-IL-4 (#554435; BD Biosciences, Franklin Lakes, NJ, USA), and APC-IL-17 (#17-7177-81; eBioscience). MitoROS were stained using MitoSOX (M36008; Thermo). The stained cells were analyzed via flow cytometry using a FACS Calibur instrument (BD Biosciences).

### Quantitative polymerase chain reaction

The mRNA expression levels of αSMA (5’-TGGGTGACGAAGCACAGAGC-3’/5’-CTTCAGGGGCAACACGAAGC-3’), COL1A1 (5’-CATAAAAGGCCCACTACCCAAC-3’/5’-ACCTTGCTCTCCTCTTACTGC-3’), fibronectin (5’-CCATAAAGGGCAACCAAGAGAGC-3’/5’-AAACCAATTCTTGGAGCAGGCG-3’), COX5b (5’-AGTCCCCTCCATCTCCAACA-3’/5’-CTGGGGCACCAGCTTGTAAT-3’), and ATP5O (5’-GAGAGGTTCTCTCCCCTCACT-3’/5’-CAAGGTACCTCTCCGCGATG-3’) were normalized to the expression of β-actin. mRNA was extracted using TRIzol (Molecular Research Center, Cincinnati, OH, USA). cDNA was synthesized using a superscript reverse transcription system (TaKaRa, Shiga, Japan). qPCR was performed using LightCycler FastStart DNA Master SYBR Green I (TaKaRa) in accordance with the manufacturer’s instructions.

### Measurement of oxygen consumption rate

OCR was quantified using an XF24 extracellular flux analyzer (Seahorse Bioscience, Chicopee, MA, USA). Splenocytes (1 × 10^6^) were placed in each well of a 96-well plate. On the following day, the cells were harvested and resuspended in XF assay media supplemented with 1 mM sodium pyruvate, 2.5 mM glucose, and 4 mM GlutaMax. Then the cells were plated in XF24-well culture microplates and incubated in a non-CO_2_ incubator for 30 min. Mitochondrial electron transport was assessed through sequential injections of 4 μM oligomycin, 3 μM carbonyl cyanide 4-(trifluoromethoxy)-phenylhydrazone, and 2 μM rotenone/2 μM antimycin A.

### Statistical analysis

The results are presented as means ± standard errors of the mean. The data were analyzed via Student’s t-test or the Mann–Whitney U test using Prism 5 software (GraphPad Inc., San Diego, CA, USA). P-values < 0.05 (two-tailed) were considered indicative of statistical significance.

## Results

### mtSTAT3 levels in the colons of UC patients are linked to disease severity

**Figure 1:**
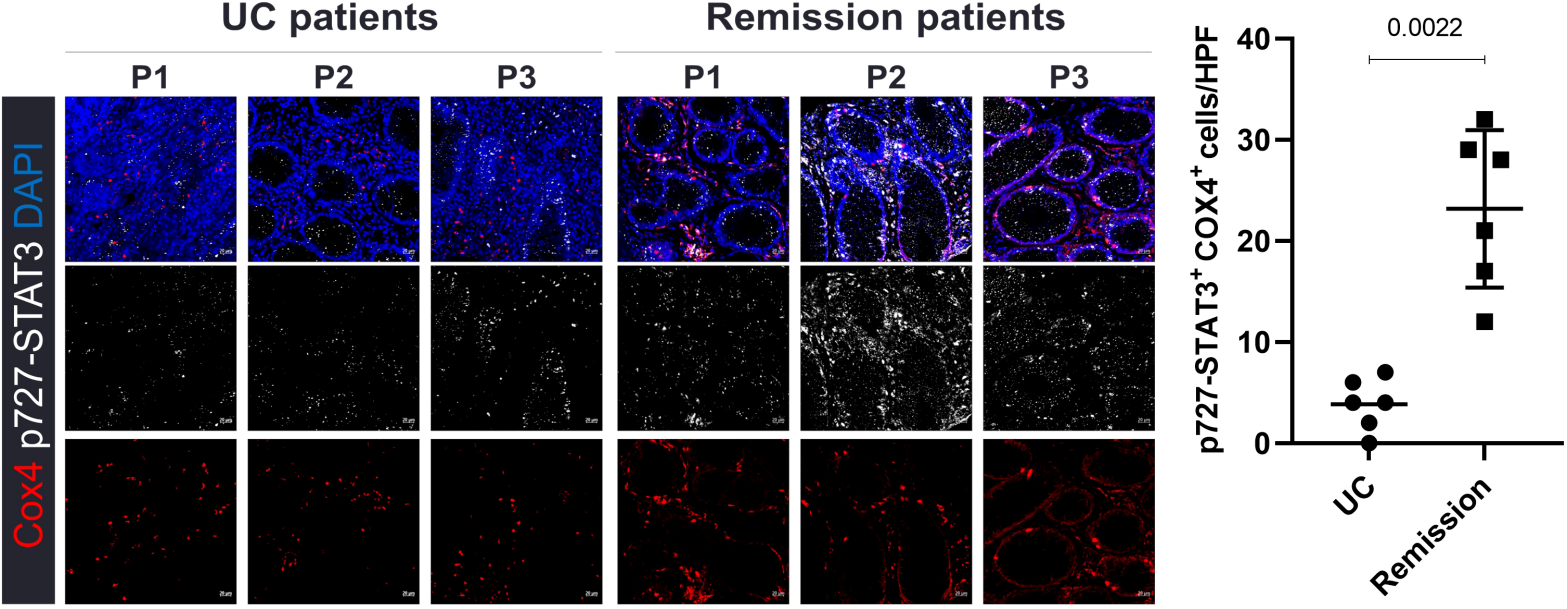
Expression of mtSTAT3 in the intestines of UC patients. Representative images of patients with severe colitis and those in remission; n = 6 patients per group.

Patients with intestinal disease have less active mitochondria in their colons. We investigated the differences in mtSTAT3 expression in the intestinal tissues of patients according to the severity of intestinal disease. Expression levels were higher in patients whose disease improved and achieved remission (**Figure 1**). There was a correlation between intestinal disease and mtSTAT3 expression.

### mtSTAT3 regulates mitochondrial function and fibrosis in human intestinal cells

**Figure 2:**
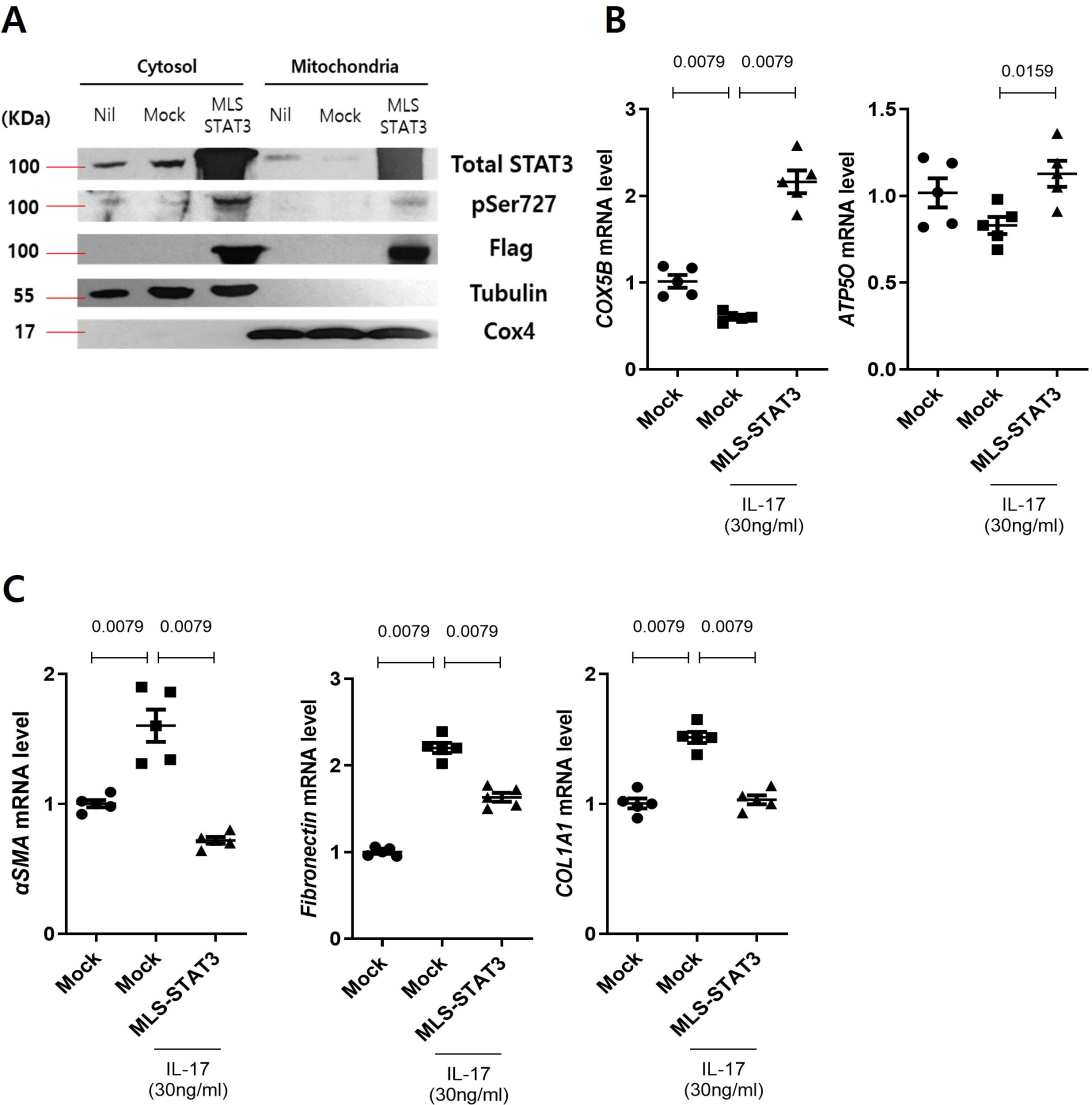
mtSTAT3 regulates mitochondrial function and fibrosis in human intestinal cells. Overexpression of mtSTAT3 reduced the fibrosis marker C18. **(A)** Lysates of C18 cells transfected with mock and mtSTAT3 overexpression vector were analyzed for mtSTAT3 protein in mitochondria by Western blotting. **(B, C)** mRNA levels of the OXPHOS complex genes COX4 and ATP5O and the fibrosis genes aSMA, fibronectin, and COL1A1 according to real-time PCR.

To ascertain whether the overexpression of mtSTAT3 helps to ameliorate IBD, we constructed an mtSTAT3 vector and evaluated its overexpression in CCD18CO (hereafter C18) cells (**Figure 2**). The vector improved the activity and efficiency of the mitochondrial OXPHOS complex in C18 cells, and decreased the levels of the fibrosis factors αSMA, fibronectin, and COL1A1.

### mtSTAT3 vector alleviates bowel inflammation in mice

**Figure 3:**
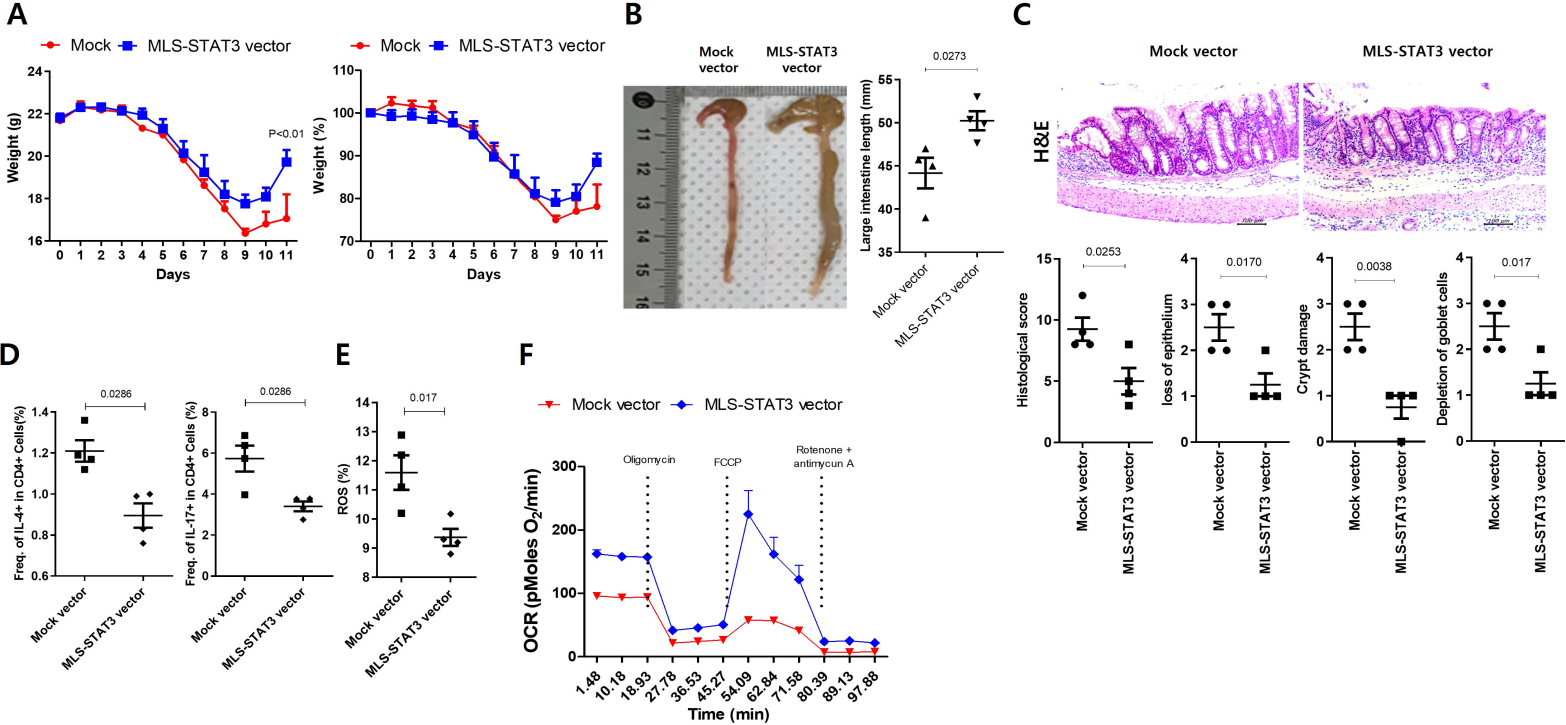
mtSTAT3 vector alleviates bowel inflammation in mice with colitis. mtSTAT3 overexpression vector reduced the susceptibility of mice to colitis. **(A)** Mice with colitis were administered mock or mtSTAT3 vectors, and their body weights were measured as a percentage of that at baseline (n = 5 per group). **(B)** Microscopic images of the colon and colon lengths. **(C)** Colon tissues of mock and mtSTAT3 vector mice were subjected to H&E staining. **(D)** Th2 and Th17 cells and **(E)** mtROS in MLNs, as revealed by flow cytometry. **(F)** Mitochondria OXPHOS metabolism in MLNs as revealed via OCR assays.

Mice with colitis treated with mock vector showed marked weight loss from day 4 as a result of severe colitis, whereas those treated with mtSTAT3 vector did not (**Figure 3**; **Supplementary Figure S1**). The latter group also showed less intestinal loss, reduced infiltration of inflammatory cells in the intestinal tissue, and less histopathological damage. Next, we assessed immune cell and mitochondrial function in mesenteric lymph nodes (MLNs) in the intestinal tissue. The activity of Th2 and Th17 cells in MLNs was reduced in the mtSTAT3 vector group; in addition, the lymph nodes displayed lower levels of mtROS and notably higher metabolic activity.

### IBD in mtSTAT3 TG mice

**Figure 4:**
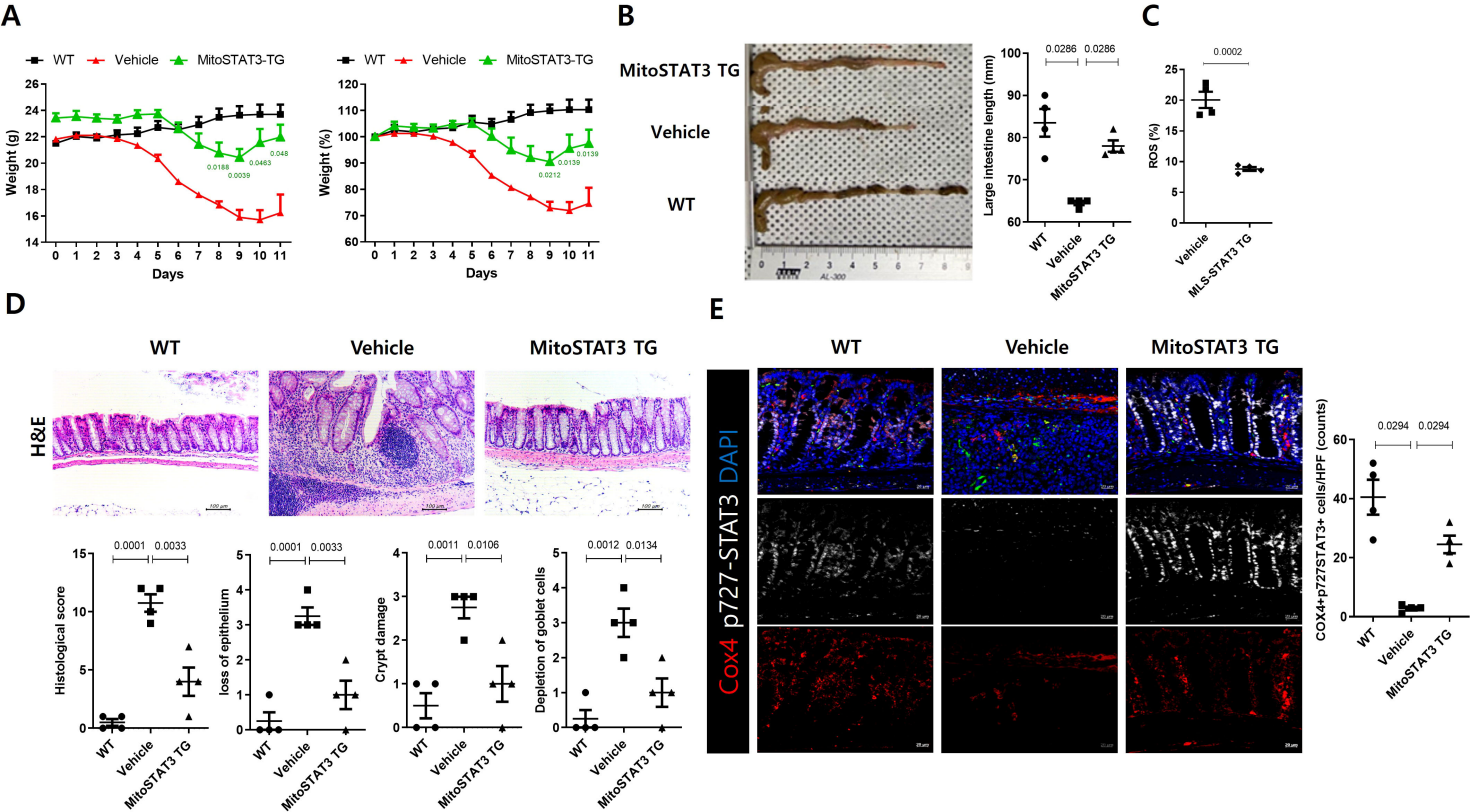
DSS-induced IBD in mtSTAT3 TG mice. Therapeutic effect of mtSTAT3 in transgenic mice. Transgenic mice overexpressing mtSTAT3 **(TG)** were generated on a C57BL/6 background. **(A)** WT and TG mice were treated with DSS, and their body weights were measured as a percentage of that at baseline (n = 5 per group). **(B)** Representative Microscopic images and colon length of each group. **(C)** Levels of mtROS in MLN cells as analyzed by flow cytometry. **(D, E)** Colon tissues in each group as revealed by H&E staining and treatment with mtSTAT3 antibodies.

To confirm the therapeutic effect of mtSTAT3, we induced colitis in wild-type and TG mice (**Figure 4**). The TG group exhibited a reduction in intestinal loss and mtROS expression relative to controls. They also had a lower incidence of intestinal inflammatory infiltration and injury, with markedly elevated levels of mtSTAT3 in intestinal tissue.

### Inflammation and fibrosis

**Figure 5:**
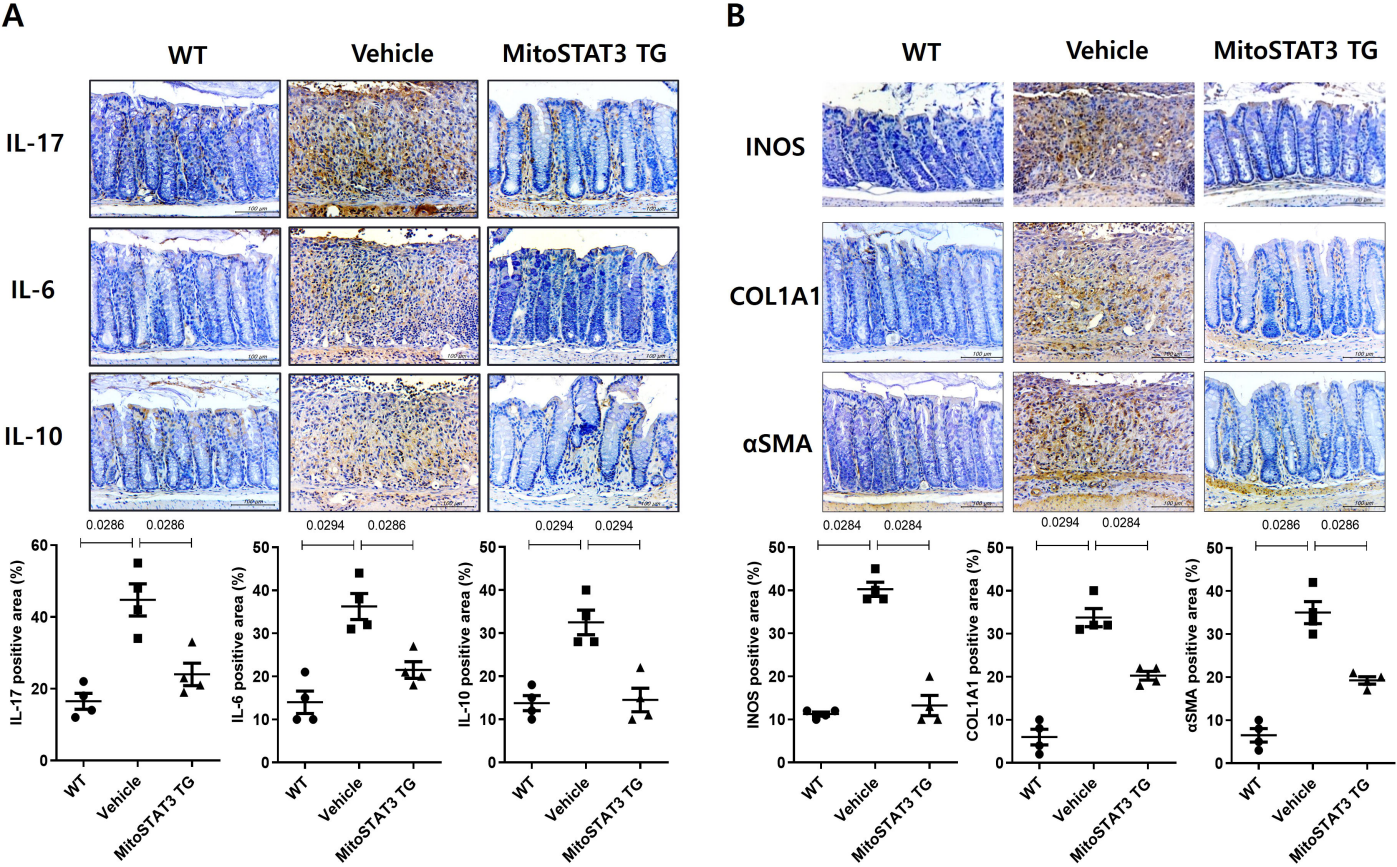
Expression of inflammatory cytokines and fibrosis markers in the intestines of TG mice. mtSTAT3 decreases inflammation and fibrosis in mice with DSS-induced colitis. **(A)** Results of immunohistochemistry using the anti-IL-17, anti-IL-6, and anti-IL-10 and **(B)** iNOS, aSMA, and collagen-1-A1 (Col1A1) antibodies. Bars are percentages of positive areas per field. Data are means ± SEMs of two replicates.

In mice with colitis, treatment with mtSTAT3 reduced the levels of inflammatory factors such as IL-17, IL-6, and IL-10; ROS markers such as iNOS; and fibrosis markers such as αSMA and COL1A1 (**Figure 5**). These results indicate that mtSTAT3 reduces inflammation and fibrosis, thereby enhancing the intestinal environment.

### Gut microbiome of TG mice

**Figure 6:**
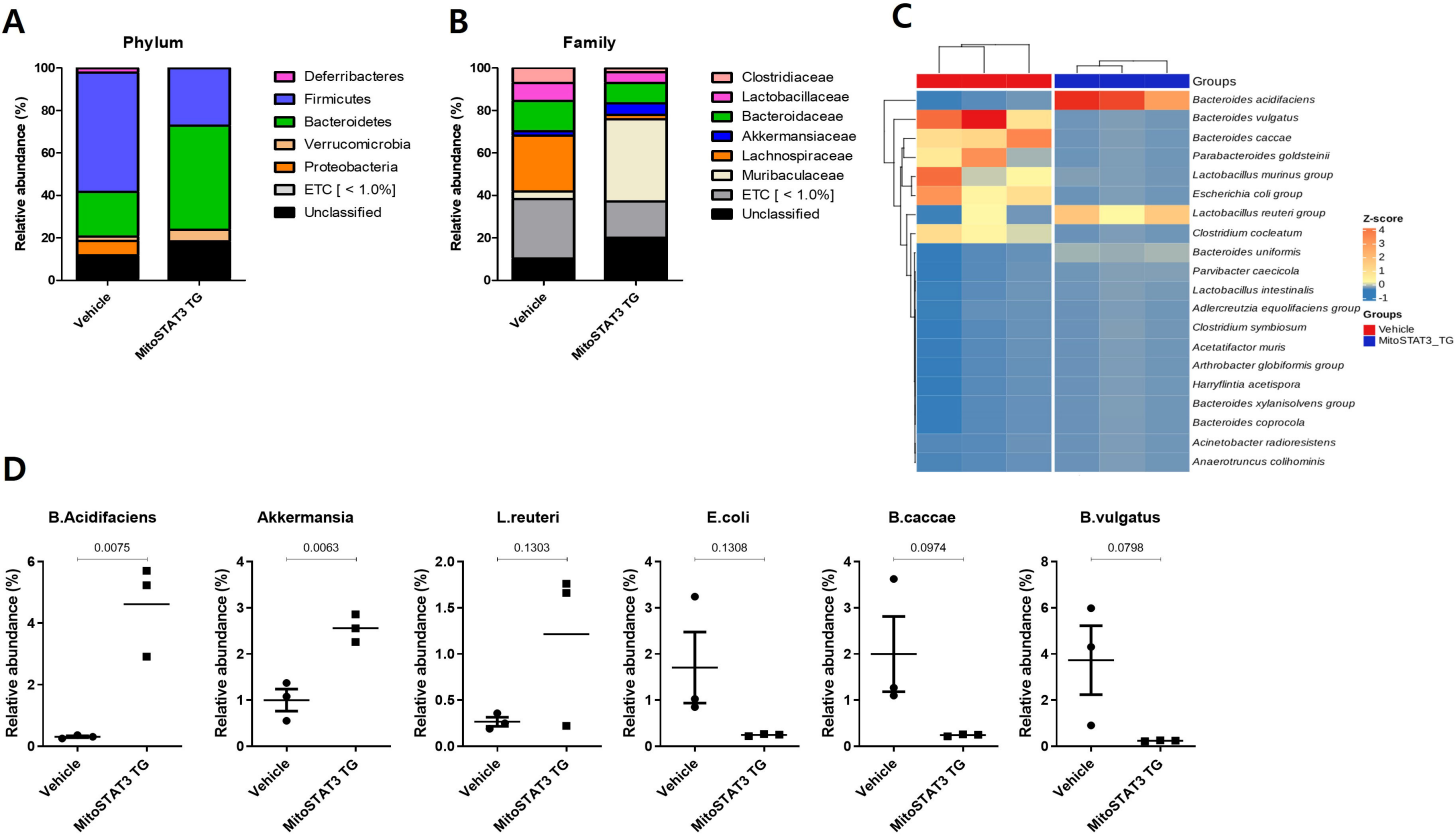
Microbiome of the guts of TG mice. The microbiome of each group was analyzed at the **(A)** phylum, **(B)** family, and **(C)** genus levels. **(D)** Microbiome heatmap, highlighting the top 20 OTUs from three fecal samples.

Next, we investigated whether mtSTAT3 is associated with changes in the gut microbiome by sequencing three TG and three control mice. The microbial composition of the gut was altered by mtSTAT3 expression (**Figure 6**). At the phylum level, the TG group had lower abundances of Firmicutes and Proteobacteria, and a higher abundance of Bacteroidetes, compared to controls. At the family level, the abundances of Clostridiaceae and Lachnospiraceae were higher in controls. A heat map was used to illustrate the community composition and normalized abundances of the microbiome based on fecal analysis. There was a reduction in the abundances of *Bacteroides vulgatus*, *Bacteroides caccae*, *Clostridium*, and *Escherichia coli* in controls, which was induced by inflammation. The TG group had larger populations of *Bacteroides acidifaciens*, which is associated with protection against liver injury, and the probiotic bacteria *Bacteroides acidifaciens*, *Akkermansia*, and *Lactobacillus reuteri*. This group also had significantly lower abundances of *E. coli*, *Bacteroides vulgatus*, and *Bacteroides caccae*.

## Discussion

Current therapeutics to treat IBD include biologics such as anti-TNF-α inhibitors, immunomodulators, aminosalicylates, and corticosteroids. However, the therapeutic outcomes vary (18). For example, only 30% of patients are primary responders, and up to 50% of patients are secondary nonresponders, to anti-TNF-α (19). In addition, fibrosis in IBD can cause several serious sequelae such as intestinal stricture or penetration, and it is thus important to develop therapeutic agents targeting it. Despite various efforts to limit intestinal fibrosis in IBD patients, current therapeutics exhibit limited efficacy (20–22). Therefore, a new paradigm for the treatment of IBD is necessary.

Whether mitochondrial dysfunction is a cause or consequence of IBD is controversial (8, 23, 24). However, several studies have indicated the former (8, 24). For example, in one study, mitochondria-targeted antioxidants prevented barrier dysfunction and resulted in a mild disease state (25). In another study, the induction of mitochondrial damage aggravated colitis in mice while inhibition of mitochondrial ROS improved it (24). Consistent with these findings regarding mitochondrial importance, we observed that mtSTAT3 expression was significantly higher in UC patients who achieved remission compared to those with severe disease. Although this clinical part of our study focused on using remission as a reference point for mucosal healing, the results suggest that mtSTAT3 has potential as a marker for therapeutic response. Building on this clinical observation. In our study, mtSTAT3 protected against IBD by enhancing mitochondrial function. This resulted in an increase in mRNA expression levels of the OXPHOS complex gene in the C18 cell line and a decrease in Th17 frequency and ROS production. Similarly, TG mice had lower levels of inflammatory cytokines than controls.

Fibrosis of the intestinal mucosa is an important complication in IBD that damages the structure and function of the intestine, which leads to a low quality of life (26, 27). Unfortunately, current treatment methods do not effectively prevent or decrease fibrosis, and IBD patients frequently undergo surgical intervention. Chronic inflammation in the gastrointestinal tract has long been considered a cause of tissue damage and remodeling, which result in fibrosis in IBD patients. In particular, Th17 cells play an important role in promoting a fibrotic phenotype in various diseases, including intestinal fibrosis (28–31). In our study, treatment with mtSTAT3 vector resulted in decreased expression of Th2 and Th17 in the lymph nodes and intestinal mucosa of mice with colitis. In addition, enhancement of mitochondrial function via mtSTAT3 led to reduced expression of fibrotic factors such as aSMA, Col1A1, and fibronectin, less shortening of the intestine, and well-maintained mucosal histology. A recent study also demonstrated that appropriate mitochondrial function is necessary for mucosal healing (32). Although it remains unclear whether mtSTAT3 directly suppresses fibrosis or does so via the Th17 pathway, our data suggest that appropriate mitochondrial function is important in preventing or alleviating intestinal fibrosis in IBD.

Significant changes in the gut microbiome of patients, and its role in the pathogenesis of IBD, have been discussed (33–35). Such changes include an increase in harmful bacteria such as Proteobacteria along with a decrease in beneficial bacteria such as *Bifidobacterium* species, *Roseburia intestinalis*, and *Fecalibacterium* (34). In our study, the microbiome of TG mice had a greater proportion of *Lactobacillus* and *Akkermansia* compared to controls. These are considered symbiotic bacteria. The modulation of *Lactobacillus* species via the administration of probiotics has shown some benefits for bowel disorders, and *Akkermansia muciniphila* is a symbiotic bacteria that resides in mucus, induces homeostatic IgG production, and controls pathobionts (36, 37). At the same time, TG mice had smaller populations of *Bacteroides*, which is common in IBD patients, where *Firmicutes* and *Bacteriodes* are dominant in healthy guts. This conflicting result might be due to the different microbiomes of humans and mice. Also, because the observed changes in the microbiomes of TG mice cannot be definitively concluded as being causes or consequences, further study of the interactions between mitochondrial function and the microbiome is needed.

While previous research has established the essential role of mtSTAT3 in maintaining epithelial homeostasis and mitochondrial respiration (38, 39), our study extends these findings by exploring the previously uncharacterized effects of mtSTAT3 on the fibrotic cascade and gut microbiome. To the best of our knowledge, this is the first study to demonstrate that mtSTAT3 exerts a direct protective effect against intestinal fibrosis and promotes the restoration of symbiotic microbiota in IBD. Overexpression of mtSTAT3 enhanced the function of mitochondria and decreased the severity of colitis by reducing inflammation and fibrosis. Although this study primarily focused on the initiation of fibrosis and myofibroblast activation through markers such as αSMA and Col1A1, further investigations into ECM degradation enzymes, such as MMPs and TIMPs, will be necessary to fully elucidate the role of mtSTAT3 in chronic tissue remodeling. Nevertheless, our results provide compelling evidence that appropriate mitochondrial function plays an essential role in IBD and suggest the possibility of therapeutic use of mtSTAT3 for IBD patients.

## Data Availability

The authors confirm that the data supporting the findings of this study are available within the article. Gut microbiome analysis was deposited in the BioProject and Sequence Read Archive (SRA; https://www.ncbi.nlm.nih.gov/sra) under the accession numbers PRJNA1205525.
