# Supplementary material for: Overexpression of mitochondrial STAT3 protein improves colonic inflammation and fibrosis in inflammatory bowel disease by enhancing mitochondrial function
Source: Front Immunol. 2026 Apr 13;17:1728341. doi: 10.3389/fimmu.2026.1728341 (PMC13111109; doi:10.3389/fimmu.2026.1728341)
Supplement: Supplementary file 1 [file Image1.pdf]

Overexpression of mitochondrial STAT3 protein improves colonic inflammation and fibrosis in inflammatory bowel disease by enhancing mitochondrial function

A Ram Lee [1,2,*], Haeyoun Choi [3,4,5,*], Seon-Yeong Lee [1,2], Hye yeon Kang [1,2,3],

Young-Mee Moon [1,2], Bo-In Lee [6], Mi-La Cho [1,2,3,#]

[1] Lab of Translational ImmunoMedicine, Catholic Research Institute of Medical Science, College of Medicine, College of Medicine, The Catholic University of Korea, Seoul, Republic of Korea

[2] Department of Pathology, College of Medicine, The Catholic University of Korea, Seoul 06591, South Korea

[3] Department of Biomedicine & Health Sciences, College of Medicine, The Catholic University of Korea, Seoul 06591, Korea

[4] Department of Microbiology, College of Medicine, The Catholic University of Korea

[5] Catholic Hematopoietic Stem Cell Bank, College of Medicine, The Catholic University of Korea

[6] Division of Gastroenterology, Department of Internal Medicine, College of Medicine, Seoul St. Mary's Hospital, The Catholic University of Korea, Seoul 06591, Korea

*These authors contributed equally to this work.

# Corresponding authors.

Correspondence to

Mi La Cho, Ph.D.
Lab of Translational ImmunoMedicine, Catholic Research Institute of Medical Science, College of Medicine, College of Medicine, The Catholic University of Korea, Seoul, Republic of Korea.

E-mail: iammila@catholic.ac.kr

Tel: +82-2-2258-7467; Fax: +82-2-599-4287

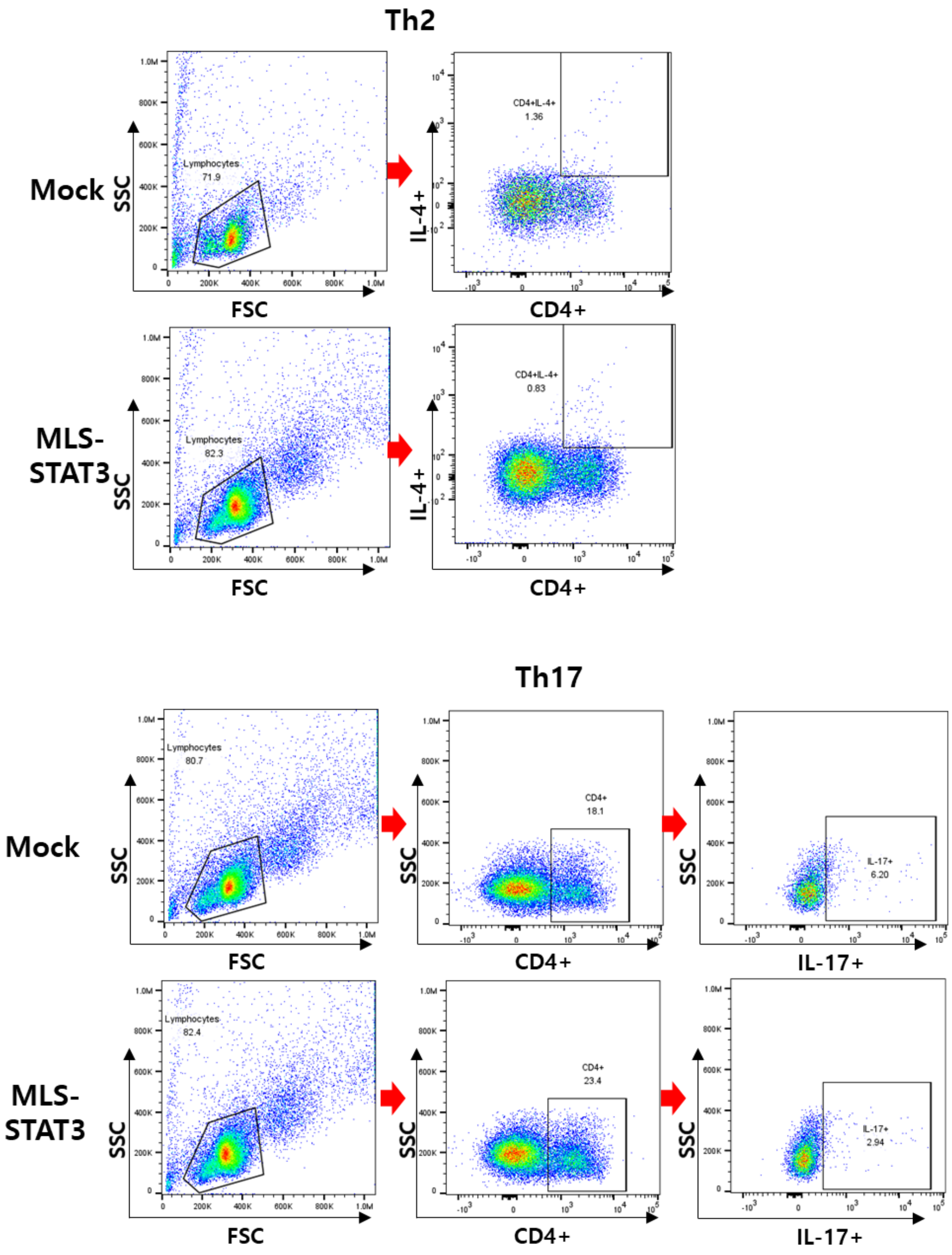

Figure S1. Flowchart of flow cytometry analysis to T_h2 and T_h17 cells. The flow cytometry picture of mesenteric lymph nodes (MLNs). The flow cytometry picture of T_h2 (IL-4+) cells and T_h17 (IL-17+) cells gated from CD4 T cells.
